# Laser-Ablated Gold Nanoparticles as Tunable Contrast Agents for Preclinical Imaging

**DOI:** 10.3390/nano15241851

**Published:** 2025-12-10

**Authors:** Yulia Finogenova, Vsevolod Skribitsky, Alexey Lipengolts, Angelina Skribitskaya, Anton Kasianov, Kristina Shpakova, Artem Laktionov, Islam Sozaev, Anna Smirnova, Elena Grigorieva

**Affiliations:** 1 N.N. Blokhin National Medical Research Center of Oncology, Moscow 115522, Russia; skvseva@yandex.ru (V.S.); a_kasianov@mail.ru (A.K.); shpakova.k.e@gmail.com (K.S.); smirn-ova@mail.ru (A.S.);; 2 Engineer and Physics Institute of Biomedicine, National Research Nuclear University MEPhI, Moscow 115409, Russia; sav1998@list.ru (A.S.); aalaktionov@mephi.ru (A.L.); islam.sozaev@mail.ru (I.S.); 3 Kurnakov Institute of General and Inorganic Chemistry, Russian Academy of Sciences, Moscow 119991, Russia; 4 The Loginov Moscow Clinical Scientific Center, Moscow 111123, Russia

**Keywords:** gold nanoparticles, contrast agents, computed tomography, biodistribution, pharmacokinetics, nanoparticle coating, tumor targeting, mammary adenocarcinoma Ca755, preclinical studies, acute toxicity

## Abstract

Femtosecond laser-ablated gold nanoparticles (AuNPs) offer a unique platform for developing novel cost-effective contrast agents due to their ultraclean, surfactant-free synthesis and precisely tunable surface properties. This study developed three computed tomography (CT) contrast agents from a single stock solution of laser-ablated AuNPs, functionalized with polyethylene glycol (PEG-2kDa, PEG-4kDa), or lipoic acid with bovine serum albumin (BSA). The primary objective was to evaluate the safety and functional efficacy of these coated AuNPs in healthy and tumor-bearing mice. After a single intravenous injection (690 ± 30 mg Au/kg), all formulations were well tolerated with no acute toxicity observed. PEGylated AuNPs demonstrated long blood half-life (18 ± 2 h for PEG-2kDa; 37 ± 2 h for PEG-4kDa), making them suitable for cardiovascular imaging up to 24 h post-injection. BSA-AuNPs had a rapid blood clearance (T_1/2_ = 2.8 ± 0.9 h), permitting cardiovascular assessment during the first 3 h, and provided intense, persistent contrast in abdominal organs, enabling liver imaging from 5 min and spleen imaging from 1 h post-injection. In a Ca755 mammary adenocarcinoma model, PEGylated AuNPs selectively accumulated in the tumor stroma and fibrous septa, allowing for precise tumor margin delineation and analysis of internal architecture. The findings establish that a single AuNP stock can be used to produce specialized contrast agents for specific imaging applications.

## 1. Introduction

Computed tomography (CT) is widely employed in biomedical studies involving small laboratory animals to address key research questions, including subject selection for experiments, longitudinal monitoring of model pathology development, and assessment of response to therapeutic intervention [1,2]. However, reliable distinguishing between internal organs on CT scans remains challenging due to the animals’ small size and the inherently low soft-tissue contrast resolution. Conventional approaches relied on manual segmentation and atlas co-registration, including articulated atlases and statistical shape models [3,4]. Recent advances utilize machine learning (ML) and artificial intelligence (AI) for automated analysis; popular options, such as AIMOS and U-Net, are based on fully convolutional networks [5,6].

Segmentation is especially complicated in experimental oncology, where it is critical not only to identify healthy organs, but also to precisely delineate implanted tumors. The tumors exhibit high variability in size, shape, and margin characteristics, and their location within the body depends entirely on the transplantation method [7]. Utilization of contrast agents significantly improves the accuracy and reliability of both manual and AI-driven segmentation [8,9]. Conventional low-molecular-weight iodinated contrast agents, designed for human clinical use, can be employed for this purpose, but their utility is limited by rapid renal excretion. To overcome this limitation, long-circulating agents have been developed specifically for application in small laboratory animals [10].

Among them are iodinated colloidal solutions such as Fenestra LC, Fenestra VC, eXIA 160, and eXIA 160 XL. These colloids are not rapidly cleared by the kidneys, enabling prolonged blood circulation, with subsequent accumulation predominantly in the liver and spleen. As all mentioned iodinated colloids are biodegradable, hepatic contrast enhancement gradually diminishes, and signal intensity returns to native levels within 3–16 days, depending on the specific agent and administered dose [11,12,13].

An alternative approach to the development long-circulating contrast agents involves the use of engineered nanoparticles [14]. The elemental composition of nanoparticles governs their X-ray attenuation properties (radiodensity), while particle size and surface coating determine biodistribution. By optimizing these parameters, nanoparticles acquire the ability to passively accumulate in subcutaneous and orthotopic tumors via the enhanced permeability and retention (EPR) effect [15,16].

Notable commercial agents in this category include ExiTron™ nano 12000 and 6000 (alkaline-earth nanoparticles, ~80–110 nm in size) and AuroVist™ (gold nanoparticles with core diameters of 1.9 nm or 15 nm). Both classes provide significantly higher hepatic contrast enhancement than iodinated emulsions [13,17]. Furthermore, novel nanoparticle-based contrast agents utilizing gold, bismuth or gadolinium are under active development [18,19,20].

Gold nanoparticles (AuNPs) offer distinct advantages, including biocompatibility, chemical inertness, and strong X-ray attenuation. They are non-biodegradable, and only particles < 5.5 nm undergo renal clearance due to glomerular filtration [21,22]. AuNPs larger than 5.5 nm have no known metabolic pathways and cannot be degraded or excreted by biological systems. Consequently, after accumulation in the liver and spleen, they persist for extended periods (up to 6 months) [23]. This makes possible repeated longitudinal imaging of abdominal organs without requiring additional contrast injections. These properties make AuNPs the preferred choice for development of preclinical contrast agents.

Strategies for AuNP synthesis are broadly divided into two categories: chemical and physical methods. The Turkevich chemical method remains the most common approach for synthesizing AuNPs in laboratory practice. Various modifications of this protocol enable the production of nanoparticles of diverse shapes and sizes (down to a very small 2–3 nm). In this method, citrate acts as a reducing agent and also as a stabilizing coating for the newly formed particles [24]. Subsequently, citrate can be replaced by another coating, the most common is polyethylene glycol (PEG).

Numerous studies have attempted to develop novel contrast agents based on nanoparticles synthesized via the Turkevich method and coated with PEG. For instance, in the study [25] ~38 nm AuNPs enabled visualization of mouse blood vessels, including those in HT-1080 tumor model, for up to 24 h after intravenous injection. In [26], ~30 nm AuNPs were administered to mice with subcutaneous A431 tumors. PEGylated AuNPs provided tumor contrast enhancement from 34 ± 5 to 78 ± 15 HU, while those additionally functionalized with an anti-EGFR monoclonal antibody reached 190 ± 12 HU. The authors of [27] investigated the size-dependent properties of PEGylated AuNPs using six variants ranging from 4 to 152 nm. Contrast properties were similar across all nanoparticle types, but their pharmacokinetics differed significantly: particles sized 4–50 nm exhibited prolonged circulation in the bloodstream, whereas those sized 79–152 nm rapidly accumulated in the liver and spleen of mice within 2 h. Thus, contrast agents based on Turkevich-synthesized nanoparticles are well-studied and thoroughly characterized, in contrast to laser-ablated nanoparticles.

Laser ablation technology yields nanoparticles of an exclusively spherical morphology. A significant advantage of this technique is that the synthesis can be conducted in pure water, resulting in a final colloid free from chemical contaminants or reaction by-products [28]. The laser ablation method is highly reproducible and allows for the rapid, large-scale synthesis of AuNPs necessary for biomedical applications. Furthermore, when transitioning to industrial-scale production, laser ablation proves more economically cost-effective than traditional chemical routes [29]. Consequently, it is a particularly suitable method for producing preclinical contrast agents. However, data supporting the use of laser-ablated nanoparticles for this purpose are very limited. The few available studies are based on planar X-ray radiography [30,31] or CT that suffers from poor image quality and a lack of temporal resolution analysis [32].

It is important to note that AuNPs produced by laser ablation are initially bare and require subsequent surface functionalization for biomedical use. Also the size of AuNPs that can be produced by laser ablation is less variable than by chemical methods. That is why pharmacokinetic properties of laser ablated AuNPs can be more effectively changed by varying coating rather than the size of the particles. Depending on the type of coating applied, the AuNPs can be engineered to possess specific and desirable pharmacokinetic properties.

The aim of this study was to fabricate three distinct contrast agents from a single stock solution of laser-ablated nanoparticles, which production is well-scalable and cost-effective, and to evaluate their safety and functional suitability as CT contrast agents in healthy and tumor-bearing mice to prove new technological concept of nanoparticles-based CT contrast agent production for scientific purposes. In general the behavior of any nonspecific contrast agents is determined by its blood circulation time. Thus, the objective of our research was to use AuNPs coatings which provide short blood circulation time for abdominal organs (liver and spleen) imaging, long blood circulating for cardiovascular imaging and extralong blood circulation time for tumor imaging. To achieve this goal we investigated three variants of nanoparticles coatings: polyethylene glycol (PEG) with molecular weights 2 kDa and 4 kDa, and lipoic acid, which was additionally stabilized with bovine serum albumin (BSA, 69 kDa). These polymers were chosen as the most studied and widely used for AuNPs coating and which potentially can change significantly pharmacokinetic behavior of AuNPs. Using computed tomography, we assessed the influence of the coating on the agents’ biodistribution, which determines their suitability for specific research applications. Obtained results also can be used as a part of validation set for ML and AI pharmacological predicting tools.

## 2. Materials and Methods

### 2.1. Laser-Ablative Synthesis of AuNPs

AuNPs were synthesized using a single-step laser ablation in liquid, following previously established protocols [33]. Briefly, a solid gold metal target (99.99%) was placed in a cuvette filled with deionized water containing 180 µM NaCl. The target was irradiated with laser pulses generated by a Yb:KGW laser (wavelength: 1030 nm, pulse duration: 250 fs, pulse energy: 30 µJ, repetition rate: 100 kHz; model TETA 10, Avesta, Moscow, Russia).

### 2.2. Functionalization of AuNPs Surface

The synthesized colloidal solution of AuNPs was centrifuged at 4255× *g* for 20 min to remove large particles. Following centrifugation, the supernatant was carefully separated from the pellet. The pellet was discarded, and the supernatant was divided into three equal portions for functionalization with different stabilizing molecules.

The first coating procedure utilized 2 kDa mPEG-SH polymer (O-(2-Mercaptoethyl)-O′-methylpolyethylene glycol, Sigma-Aldrich, St. Louis, MO, USA). The detailed functionalization protocol is described in [34]. Briefly, we determined the size and concentration of gold nanoparticles in the solution using spectrophotometric method according to [35]. The total surface area of nanoparticles was calculated, and 2 kDa mPEG-SH polymer was added at a ratio of two polymer molecules per 1 nm^2^ of nanoparticle surface area.

For the second coating, a 4 kDa PEG-lipoic acid (PEG-LA) reagent was synthesized in advance, using general approach described in [36]. Briefly, lipoic acid (1 equiv), EDC (1 equiv), and DMAP (0.05 equiv) were each dissolved in separate vials containing 1 mL of dichloromethane (DCM). The solutions were then combined in a three-neck flask under stirring. The mixture was degassed with argon for 1 h at 30 °C using a water bath. Subsequently, the mixture was cooled to 0 °C in an ice bath, and 4 kDa PEG (1 equiv), dissolved in 5 mL of DCM, was added. The reaction was stirred at 0 °C for 1 h and then allowed to proceed for 20 h at room temperature. The following extraction step involved mixing the reaction mixture with an equal volume of water. The mixture was shaken vigorously and then centrifuged at 4255× *g* for 1 min. The water phase was discarded, and this process was repeated 4–5 times until a completely transparent organic phase was obtained. Residual water was removed using a rotary evaporator at 45 °C for 40 min. The mixture was then cooled for 15 min, resulting in the formation of yellowish crystals. The crystals were dissolved in 4 mL of DCM. The final step was a reprecipitation using diethyl ether: 4 mL of the DCM solution was added to 50 mL of diethyl ether, followed by centrifugation. The ether supernatant was decanted, and the pellet was left to dry for 24 h. Finally, the coating was performed based on the principle of two 4 kDa PEG-LA molecules per 1 nm^2^ of nanoparticle surface area in the colloidal solution.

For the third coating, lipoic acid (LA) was conjugated to the nanoparticles. A quantity of lipoic acid was prepared based on the principle of two molecules per 1 nm^2^ of the AuNP surface area in the colloidal solution. This aliquot was dissolved in 1 mL of ethanol, and the solution’s pH was adjusted to 11 by titration with NaOH. The resulting lipoic acid solution was then added to the colloidal AuNP solution dropwise (one drop every 10–15 s) under constant stirring. Coating of AuNPs solely with lipoic acid did not provide AuNPs colloid stability in solutions with concentrations of gold necessary for CT imaging (>20 mg Au/mL). Subsequently, a quantity of BSA, calculated at 1:1 molar ratio with lipoic acid, was dissolved in 1 mL of deionized water and added to the colloidal gold nanoparticle solution under stirring to improve colloidal stability of the solution while concentrating.

The solutions of all the three AuNPs underwent gel-filtration with desalting column PD-10 containing Sephadex G-25M sorbent (Pharmacia & Upjohn, Stockholm, Sweden) to remove sodium chloride.

The final solutions were concentrated using a rotary evaporator and sterilized through a 0.22 μm filter.

### 2.3. Characterization of AuNPs

The characterization of the final AuNPs colloid was performed using spectrophotometric analysis, dynamic light scattering (DLS), inductively coupled plasma optical emission spectrometry (ICP-OES), scanning electron microscopy (SEM), energy-dispersive X-ray spectroscopy (EDX) and Fourier-transform infrared spectroscopy (FT-IR).

AuNPs core diameter was controlled by two methods, i.e., SEM and spectrophotometry. After removal of large particles from original AuNPs solution with centrifugation the size distribution of AuNPs was evaluated with SEM. The further control of AuNPs core size during and after functionalization was performed with spectrophotometric.

SEM imaging was conducted using a MAIA 3 electron microscope (TESCAN, Brno, Czech Republic) to obtain micrographs of the nanoparticles, which were then used to determine the gold core size.

Spectrophotometric analysis was carried out with a Cary 50 spectrophotometer (Varian, Palo Alto, CA, USA) operating in the ultraviolet and visible range. Absorption spectra of the colloidal solutions were recorded from 800 to 200 nm. These spectra were used to determine the AuNP core size and the gold concentration in the solution, in accordance with [35].

DLS and zeta potential measurements were performed using a Zetasizer Nano ZS spectrometer (Malvern Instruments, Malvern, UK). This technique was used to determine the hydrodynamic diameter of the nanoparticles in solution and to measure their zeta potential.

ICP-OES analysis was conducted using a high-resolution PlasmaQuant 9100 Series spectrometer (Analytik Jena, Jena, Germany). This method was employed to determine the elemental gold concentration in the colloidal solutions. Prior to analysis, the samples were digested in a mixture of concentrated nitric and hydrochloric acids in a 1:3 ratio (aqua regia).

EDX was performed to confirm the elemental composition of the nanoparticles’ core. It was performed using the X-Act energy-dispersive X-ray spectroscopy (EDX) module (Oxford Instruments, Oxford, UK). EDX spectra were collected at 20 keV. Samples (2 μL of colloidal solution) were placed on a silicon substrate and allowed to dry under ambient conditions.

To confirm the coating of the nanoparticles with the polymers FT-IR was performed on an IR spectrometer FT-08 Infralum (Lumex, St-Petersburg, Russia) in the range of 4000–600 cm^−1^ with a resolution of 1 cm^−1^. IR spectra were recorded as KBr pellets with a substance content of 2 wt%.

### 2.4. CT Imaging

Computed tomography of mice was performed using a VECTOR 6 preclinical trimodal scanner (MiLabs, Utrecht, The Netherlands). During scanning, animals were maintained under anesthesia induced by a 2% isoflurane-air mixture. Animal physiological status was monitored throughout the procedure using a respiratory sensor and the BioVet Rev. 04 program (Polarean Inc., Cleveland, OH, USA).

For “Total body” mode, the following parameters were used: X-ray tube voltage of 55 kV, tube current of 0.21 mA, exposure time per projection of 75 ms, and a rotation step of 0.5° with 1 projection acquired per step. The images were reconstructed with an isotropic voxel size of 80 µm.

For “Ultra focus” mode, the parameters were: X-ray tube voltage of 55 kV, tube current of 0.21 mA, exposure time per projection of 75 ms, and a rotation step of 0.15° with 2 projections acquired per step. The images were reconstructed with an isotropic voxel size of 60 µm.

Image reconstruction was performed using MiLabs Rec 12.00 software (MiLabs, Utrecht, The Netherlands). Post-processing and image analysis were conducted using PMOD 4.205 software (PMOD Technologies LLC, Zurich, Switzerland).

### 2.5. CT Radiodensity Calibration

A phantom study was performed to plot the calibration curve. For this purpose, a phantom containing a water-filled tube and tubes with colloidal AuNPs at known concentrations (1–25 mg Au/mL) was CT-scanned. The actual gold concentrations were verified by ICP-OES using a high-resolution PlasmaQuant 9100 Series spectrometer (Analytik Jena, Jena, Germany). Prior to analysis, the samples were digested in a mixture of concentrated nitric and hydrochloric acids at a 1:3 (*v*/*v*) ratio (aqua regia). Using PMOD software (PMOD Technologies LLC, Zurich, Switzerland), the radiodensity (HU) within each tube was quantified, and the radiodensity of pure water (HU) was subtracted from it. This procedure established a calibration curve plotting the increase in radiodensity (ΔHU) against gold concentration (Figure S1). The dependence of ΔHU on the gold concentration ([Au], mg/mL) was fitted with a linear function. The linear equation was:

ΔHU = 46.9 × [Au](1)

### 2.6. Contrast-Enhanced CT Imaging of Healthy Mice

All procedures involving animals were performed in concordance with local ethic regulations and approved by institutional ethic committee (No. 06a-p-2024 issued on 17 September 2024).

Healthy female BALB/c mice were divided into 3 experimental groups (*n* = 5) and 1 control group (*n* = 5). Experimental groups received a single intravenous injection of AuNPs with distinct surface coatings: PEG-4kDa, PEG-2kDa, or lipoic acid with BSA, at a dose of 690 ± 30 mg Au/kg (~15 mg Au per animal).

CT scanning was performed in “Total body” mode before injection (baseline) and at the following time points post injection: 5 min; 1, 5, 24, 48, 72 h; 4, 7, 14, 21 days; and 1, 2, 3, 4, 5, 6 months.

On the acquired images, manual segmentation of the following organs was performed: heart (left ventricle), liver, spleen, kidneys, brain, and muscle tissue of the left hind limb. The resulting HU values were converted to mg Au/mL using a calibration curve generated from phantom studies (Figure S1).

### 2.7. Tumor Uptake Evaluation

Female C57Bl/6 mice were used for this study. Murine mammary adenocarcinoma Ca755, received from tumor collection of N.N. Blokhin National Medical Center of Oncology, was transplanted subcutaneously into the right hind leg using 0.2 mL of a freshly prepared 7% (*w*/*v*) tumor cell suspension. Tumor-bearing mice were divided into 2 experimental groups and received an intravenous injection of either PEG-4kDa AuNPs (*n* = 5) or BSA-AuNPs (*n* = 5) at a dose of 680 ± 30 mg Au/kg (~15 mg Au per animal).

CT imaging was performed before injection (baseline), and at the following time points post injection: 5 min, 1, 3, 5, 24, 48, and 72 h, and 6 days. All scans were acquired in two modes: “Total Body” and “Ultra Focus”. In the “Ultra Focus” mode, the field of view was restricted to the tumor region.

Mice were sacrificed 6 days post-CT imaging by isoflurane overdose. Tumor tissue samples were collected and fixed in 10% neutral buffered formalin (volume ratio 10:1) for 24 h at room temperature. The samples were then processed in a histoprocessor STP-120 (Epredia, Portsmouth, NH, USA), embedded in paraffin blocks with TES99 system (Medite, Burgdorf, Germany), and sectioned into 2–5 μm slices using a microtome CUT6062 (Slee, Germany). Sections were mounted on glass slides, dried, and stained with hematoxylin and eosin using an automated stainer Gemini AS (Thermo Fisher Scientific, Waltham, MA, USA). Finally, the slides were coverslipped and examined by light microscopy with microscope BX46 (Olympus, Hachioji, Japan).

## 3. Results

### 3.1. Characterization and Functionalization of AuNPs

**Scheme 1:**
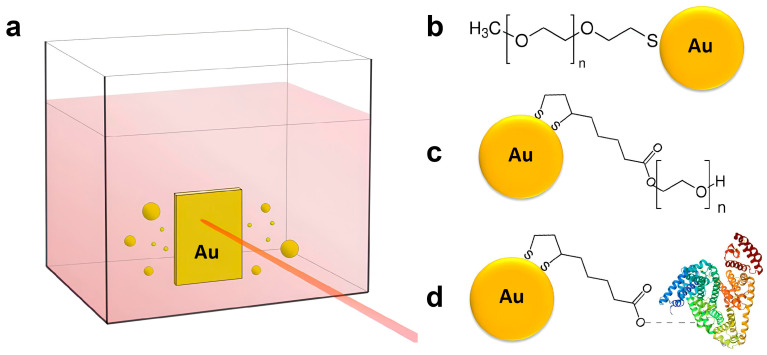
Synthesis and coating of AuNPs: (**a**) laser ablation method of AuNPs production; (**b**) schematic diagram of PEG-2kDa AuNPs coated via thiol group; (**c**) schematic diagram of PEG-4kDa AuNP coated via lipoic acid; (**d**) schematic diagram of AuNPs coated with lipoic acid and stabilized with BSA.

AuNPs utilized in this study were produced using an ultraclean synthesis approach based on femtosecond (fs) laser ablation, enabling the generation of surfactant-free colloids. Laser ablation technique is detailed in the Methods Section and schematically illustrated in Scheme 1a.

Results of EDX analysis confirm that obtained laser ablated nanoparticles comprised gold only (Figure S2).

To improve colloidal stability under physiological conditions and minimize immune recognition, AuNPs were stabilized with polymers surface coatings. Three options were used: PEG-2kDa via the thiol (-SH) group; PEG-4kDa via lipoic acid linker; lipoic acid with bovine serum albumin (BSA) as colloidal stabilizer (Scheme 1b–d). BSA was additionally added to the third formulation to improve its colloidal stability as AuNPs coated exclusively with lipoic acid were aggregating while concentrating with evaporation.

FT-IR spectra (Figure S3) of all three batched of the coated with different polymer AuNPs confirm attachment of corresponding polymer onto the AuNPs surface. The signals typically corresponded to the values reported in the literature [36,37,38].

**Figure 1:**
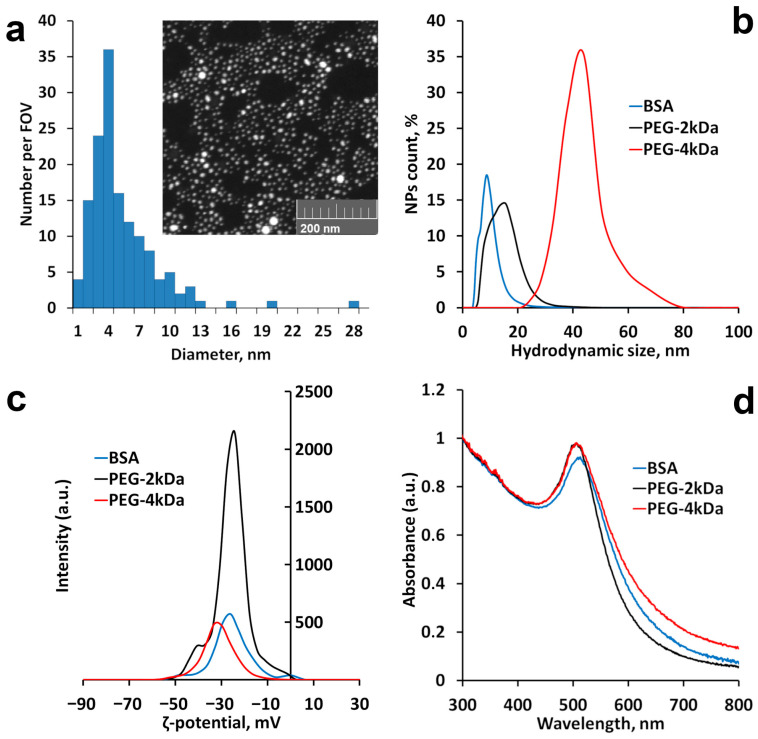
Characterization of AuNPs: (**a**) nanoparticles size distribution in stock solution according to SEM; (**b**) hydrodynamic size distribution; (**c**) ζ-potential distribution; (**d**) UV-vis spectra.

Nanoparticles’ core diameter distribution in the stock solution after centrifugation assessed with SEM is presented in Figure 1a. The distribution is slightly biased and has a low portion of tail up to 13 nm. The mean diameter of the nanoparticles is 5 ± 3. After functionalization all AuNPs had core diameter from 5.0 ± 1.3 to 6.0 ± 1.4 nm according to UV-vis spectroscopy, but hydrodynamic size differed from 9 ± 7 nm for BSA-AuNPs to 41 ± 18 nm for PEG-4kDa AuNPs (Table 1, Figure 1b). Hydrodynamic diameters distribution of stock uncoated AuNPs and coated with lipoic acid without BSA stabilization are presented in Figure S4. SEM images of AuNPs after coating are presented in Figure S5. Regardless of coating, all AuNPs had negative ζ-potential (Table 1, Figure 1c). Plasmon resonance peak position was similar for all AuNPs: 506 nm for PEGylated AuNPs and 508 nm for BSA-AuNPs (Table 1, Figure 1d). Concentration of gold in all colloidal solutions measured by UV-vis spectroscopy was 75 ± 9 mg Au/mL.

### 3.2. Acute Toxicity

Healthy BALB/c mice were divided into 3 experimental groups (*n* = 5) and 1 control group (*n* = 5). Experimental groups were administered a single intravenous injection of AuNPs with distinct surface coating: PEG-4kDa, PEG-2kDa or BSA, at a dose of 690 ± 30 mg Au/kg (~15 mg Au per animal). The injection was well tolerated by all animals, with no evidence of acute toxicity regardless of the type of AuNPs. Mice displayed normal behavior post-injection, with no indications of discomfort or neurological impairment, and no mortality was observed in the treated animals.

A rapid dark blue discoloration of the skin developed shortly after injection in all treated groups, aligning with prior reports [17,39], and did not adversely affect the animals’ condition or activity (Figure S6).

To further evaluate short-term physiological effects, body weight was monitored in 3 treated and 1 control groups over a 21-day observation period, revealing no significant differences in weight trends between groups. Measurements of daily water and food consumption also remained consistent. Collectively, these data confirm that high-dose intravenous administration of coated AuNPs was well tolerated in laboratory mice, producing no acute toxic effects despite skin discoloration.

### 3.3. Contrast Enhancement in Vivo

The functional efficacy of the AuNPs as contrast agents was evaluated in the same three mouse groups used for the acute toxicity assessment. CT imaging was performed at baseline (pre-injection) and serially at designated timepoints post-injection.

**Figure 2:**
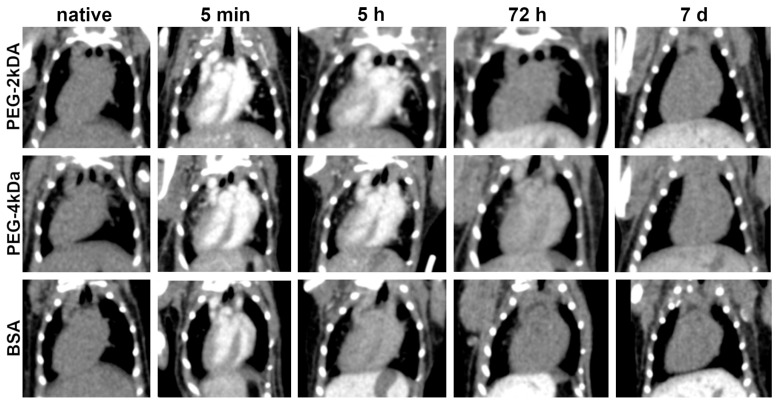
Dynamic CT images of the heart (coronal view) before and after administration of PEG-2kDa AuNPs, PEG-4kDa AuNPs and BSA-AuNPs.

Figure 2 illustrates dynamics of murine cardiac CT images from 5 min to 7 days post-intravenous AuNPs administration. On pre-injection scans, the heart exhibits radiodensity typical of soft tissues. Immediately after injection, high blood gold concentration results in intense contrast enhancement of cardiac chambers without myocardial or interventricular septum enhancement. Subsequent reduction in chamber enhancement correlates with decreasing blood gold levels, though the decline rate varied significantly by nanoparticle coating. For PEG-2kDa AuNPs, cardiac enhancement persisted at 5 h and 24 h, resolving to baseline by 48 h. PEG-4kDa AuNPs demonstrated prolonged blood circulation: the interventricular septum remained visible at 48 h, indicating ongoing nanoparticle presence. BSA-AuNPs showed the shortest circulation: septal visualization was faint at 5 h, with cardiac radiodensity returning to baseline by 24 h. By day 7 post-injection, cardiac radiodensity returned to baseline levels in all animals across groups and remained unchanged in subsequent scans.

**Figure 3:**
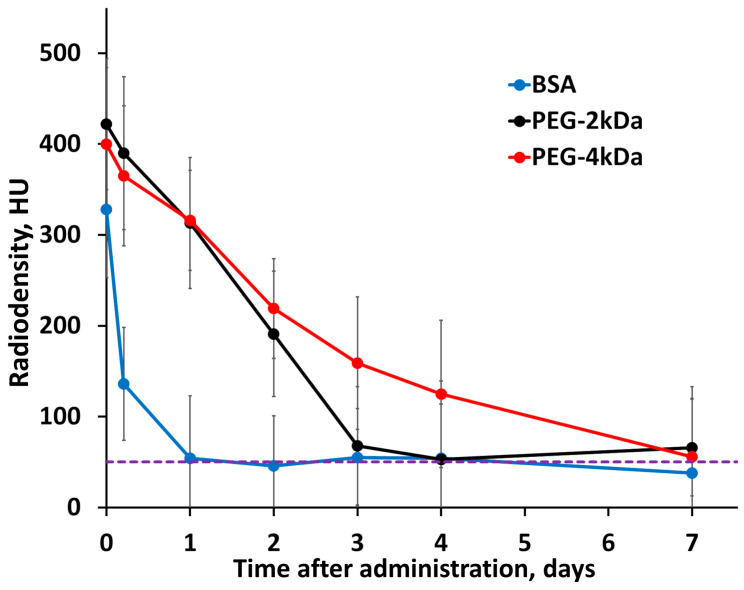
Dynamics of radiodensity (HU) in cardiac chambers post injection of AuNPs with three distinct coatings. The native radiodensity is indicated by a horizontal purple dotted line.

Dynamic cardiac radiodensity (HU) is presented in Figure 3, with the corresponding blood gold concentration (mg/mL) derived from these measurements shown in Figure S7a. This quantitative conversion is based on a predetermined linear calibration curve (ΔHU vs. mg Au/mL), which was established through prior phantom experiments. The blood half-lives of the nanoparticles, calculated from this concentration data, were as follows: PEG-4kDa AuNPs, T_1/2_ = 37 ± 2 h; PEG-2kDa AuNPs, T_1/2_ = 18 ± 2 h; and BSA-AuNPs, T_1/2_ = 2.8 ± 0.9 h. These differences in half-life directly reflect the influence of surface coating on pharmacokinetics: BSA-AuNPs are rapidly cleared from the bloodstream, while PEG coating prolongs circulation, with the longer PEG-4kDa chain providing advantage over PEG-2kDa.

**Figure 4:**
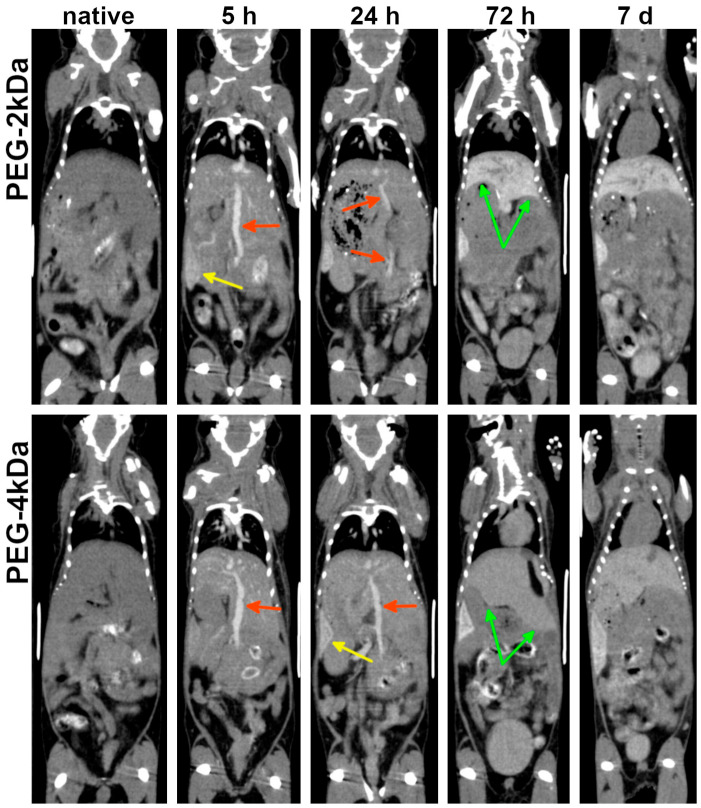
Dynamic CT images of liver and spleen (coronal view) before and after administration of PEG-2kDa AuNPs and PEG-4kDa AuNPs. Red arrows indicate blood vessels, yellow arrow—the spleen, green arrows—margin of the liver.

As blood gold levels declined, progressive gold accumulation was observed in the liver and spleen—organs abundant in mononuclear phagocytes. Both the kinetics and peak gold content in the liver were strongly dependent on nanoparticle surface functionalization. PEG-2kDa AuNPs exhibited more pronounced hepatic sequestration than PEG-4kDa AuNPs: at 72 h post-injection, liver parenchyma demonstrated marked contrast enhancement with clearly demarcated margins. Conversely, PEG-4kDa AuNPs showed negligible hepatic contrast at this timepoint (Figure 4).

Splenic accumulation onset differed substantially, with PEG-2kDa AuNPs producing distinct splenic contrast from 5 h and PEG-4kDa AuNPs—only from 24 h (Figure 4). The inverse correlation between blood circulation time and hepatosplenic uptake kinetics reflects the dominant clearance pathway—phagocytosis by hepatic and splenic macrophages. Critically, both formulations maintained detectable blood circulation for ≤24 h, permitting cardiovascular assessment during this period.

**Figure 5:**
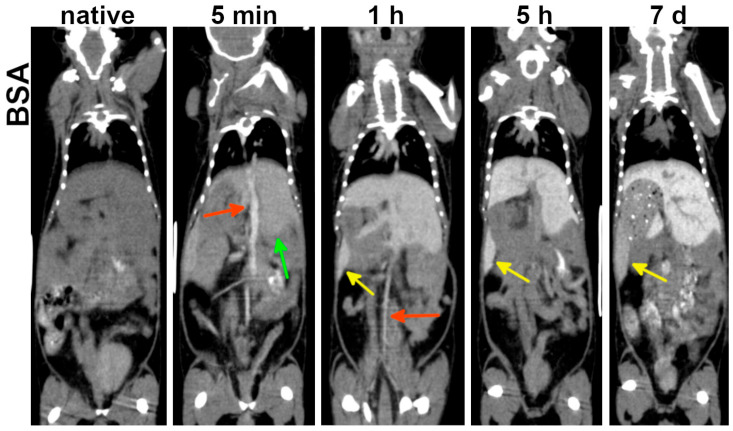
Dynamic CT images of liver and spleen (coronal view) before and after administration of BSA-AuNPs. Red arrows indicate blood vessels, yellow arrows—the spleen, green arrow—margin of the liver.

BSA-AuNPs demonstrated rapid hepatosplenic accumulation: hepatic enhancement was evident as early as 5 min post-administration, followed by pronounced splenic enhancement at 1 h (Figure 5). Due to short blood circulation time, BSA-AuNPs permitted cardiovascular assessment during the first 3 h post-injection. Beyond 5 h, only abdominal blood vessels devoid of contrast agent became discernible against the enhanced hepatic background. Thus, BSA-AuNPs offer a narrower temporal interval for cardiovascular imaging compared to PEG-coated variants, but enable earlier abdominal organ imaging.

**Figure 6:**
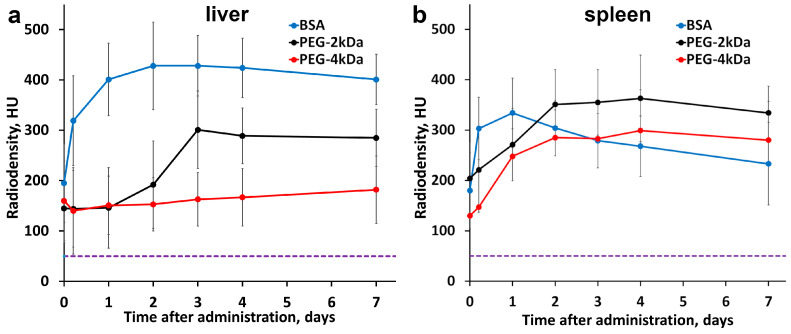
Dynamics of radiodensity (HU) in the liver (**a**) and spleen (**b**) post injection of AuNPs with three distinct coatings. The native radiodensity is indicated by a horizontal purple dotted line.

According to HU plots, maximum contrast enhancement in the liver and spleen for BSA-LA AuNPs was observed at 48 h post-administration, followed by a modest decrease in the spleen by day 7 and stable enhancement in the liver (Figure 6). Corresponding tissue gold concentrations are shown in Figure S7b,c. Comparative analysis revealed that BSA-AuNPs accumulated more in the liver than in the spleen, while PEG-4kDa AuNPs showed higher accumulation in the spleen compared to the liver. PEG-2kDa AuNPs showed similar uptake in both organs. These data conclusively demonstrate the influence of nanoparticle coating on biodistribution.

No discernible contrast enhancement was observed in the kidneys. Quantitative analysis revealed a transient radiodensity increase peaking at 30 min–1 h post-injection to +80 ± 18 HU relative to baseline values, equivalent to 1.5 ± 0.2 mg/cm^3^ of gold. Subsequently, gold concentrations in renal parenchyma declined, returning to baseline by 24 h for BSA-AuNPs and by 7 days for both PEG-AuNPs—consistent with the prolonged blood circulation of PEGylated nanoparticles.

Slight enhancement was noted in proximal ureters from 30 min to 5 h post-injection (Figure S8a). While this may indicate minor nanoparticle passage into urine, no radiodensity increase was observed in the bladder lumen at any timepoint (Figure S8b). Thus, urinary excretion cannot be considered a significant elimination pathway for the studied nanoparticles.

In general, following intravenous administration, AuNPs demonstrated blood circulation of variable duration, enhancing cardiac chambers and vasculature. A progressive signal increase was detected in the liver and spleen, while renal gold retention remained transient and below visually discernible enhancement thresholds. No AuNP uptake was detected in gastric/intestinal walls, pancreas, brain, or muscle. By day 7, all groups exhibited a stable biodistribution pattern, characterized by complete bloodpool clearance and persistent hepatosplenic accumulation. Subsequent CT scans confirmed the contrast pattern remained unchanged.

Following the initial 7-day period, animals underwent weekly CT scans (days 7, 14, 21) and then monthly CT scans for 6 months, which confirmed no substantial changes in the established biodistribution patterns (Figure S9). AuNP-derived enhancement remained exclusively localized to the liver and spleen, with no detectable signal in other organs. Quantitative analysis of the liver showed distinct retention kinetics: PEG-4kDa AuNPs maintained stable concentrations (±3%), PEG-2kDa AuNPs decreased slightly (average: −7% by 6 months relative to day 7), and BSA-AuNPs declined moderately (average: −35% by 6 months relative to day 7). The sustained hepatosplenic retention combined with total absence of bladder enhancement supports negligible renal excretion.

Potential nanoparticle toxicity was also evaluated via serial CT assessment of organ size and contours. No significant pathological changes were observed: liver maintained stable dimensions without contour nodularity, no ascites or free peritoneal fluid was detected. These findings indirectly indicate preserved hepatic function. Notably, nanoparticle-induced skin discoloration (dark blue) persisted throughout the 6-month observation period. However, standard veterinary assessments and behavioral monitoring revealed no signs of systemic toxicity.

### 3.4. Ca755 Tumor Uptake

The final experimental phase assessed AuNPs accumulation in subcutaneous Ca755 tumors in syngeneic C57Bl/6 mice. Tumor-bearing mice were injected with either PEG-4kDa AuNPs (*n* = 5) or BSA-AuNPs (*n* = 5) at a dose of 680 ± 30 mg Au/kg (~15 mg Au per animal). PEG-2kDa AuNPs were not used for tumor uptake study because of its shorter blood circulation time compared to PEG-4kDa AuNPs. CT imaging of the animals following intravenous AuNPs administration was performed in “Ultra Focus” mode, focused specifically on the tumor region, and additionally in “Total body” mode. Images were acquired at multiple time points from 5 min to 6 days post-injection.

**Figure 7:**
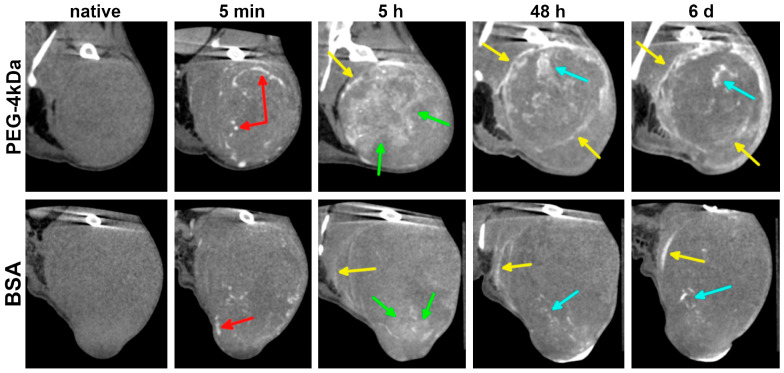
Dynamic CT images of Ca755 tumor (coronal view) before and after administration of PEG-4kDa AuNPs and BSA-AuNPs. Red arrows indicate enhanced blood vessels, green arrows—enhanced parenchyma, yellow—stromal component including tumor margins and fibrous septa, light blue—the most radiodense foci.

Within the first 5 min to 1 h, AuNPs remained predominantly intravascular, enhancing tumor blood vessels without significant differences between PEG-4kDa and BSA-AuNPs (Figure 7). By 3–5 h, PEG-4kDa AuNPs exhibited extensive extravasation into tumor parenchyma, generating heterogeneous intratumoral enhancement characterized by moderately contrasted regions interspersed with intensely radiodense foci and sharply demarcated tumor margins. This enhancement pattern evolved considerably by 48 h post-injection. While the parenchymal contrast gradually declined from its peak at 5 h, the stromal components became increasingly prominent. At 48 h post-injection PEG-4kDa AuNP-treated tumors exhibited a highly heterogeneous architecture, featuring regions of baseline radiodensity, residual moderately enhanced parenchymal zones, and well-defined, contrast-outlined fibrous septa. Enhancement of tumor stroma largely persisted through 6 days, defining the long-term retention pattern.

In contrast, BSA-AuNPs showed only faint peripheral enhancement and sparse foci at 3–5 h post-injection, with parenchymal contrast remaining minimal. This pattern did not evolve significantly; after 48 h, BSA-AuNPs demonstrated no discernible parenchymal enhancement, with only sparse foci and faint septa visible against a background of native tissue density. This is consistent with the rapid sequestration of BSA-AuNPs by the liver, which limited their blood circulation time and reduced the potential for effective tumor accumulation. Over the 6-day observation period, no further changes in the BSA-AuNP enhancement pattern were observed.

**Figure 8:**
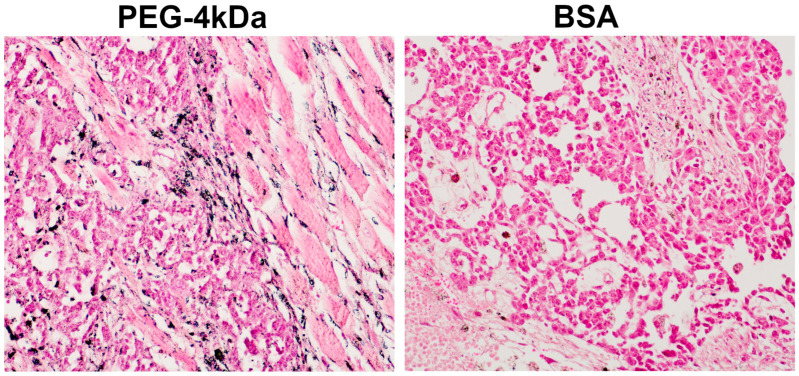
Histological sections of the Ca755 tumor obtained from mice 6 days post administration of PEG-4kDa AuNPs and BSA-AuNPs. H&E, ×200.

Immediately following the 6-day CT scan, mice were euthanized for tumor histology. Analysis revealed predominant AuNP localization within stromal compartments (Figure 8), with extensive accumulation in fibrous septa—particularly in macrophage cytoplasm and perivascular spaces—indicating active phagocytosis and stromal retention. Neoplastic parenchyma showed minimal uptake, exhibiting only sporadic cytoplasmic granules in isolated tumor cells. This limited penetration likely reflects restricted interstitial transport through the tumor microenvironment. Histopathologic findings confirmed that contrast-enhanced linear structures on CT images corresponded to fibrous septa within the tumor stroma.

## 4. Discussion

This study investigated the contrast properties of three promising AuNP-based agents for preclinical CT. The AuNPs demonstrated high radiodensity, enabling clear visualization of the cardiovascular system, liver, spleen, and subcutaneous tumor model throughout a series of scans.

A key innovative aspect of our approach lies in the use of a single stock solution of laser-ablated AuNPs, functionalized with three distinct coatings: polyethylene glycol (PEG) of two molecular weights and lipoic acid with bovine serum albumin (BSA). Since the gold core was identical for all prepared agents, the observed differences in their pharmacokinetics and biodistribution can be attributed to the influence of the surface coating. This work utilized two types of PEG-coated nanoparticles: PEG-2kDa, attached to the gold surface via an SH-group, and PEG-4kDa, conjugated using lipoic acid as a linker. We propose that the type of chemical linkage is not the primary factor affecting the biodistribution pattern, and the observed pharmacokinetic differences are determined by the length of the PEG chains.

While the influence of coating on nanoparticle behavior in vivo has been extensively investigated, all prior research has been conducted on particles synthesized by chemical methods. Specifically, studies using nanorods have demonstrated that PEG coating provides longer blood circulation time than BSA [40]. This difference can be attributed to albumin being a natural protein with established metabolic pathways and utilization mechanisms. In contrast, PEG is a synthetic inert polymer which lacks specific recognition mechanisms by immune cells.

When comparing laser-ablated nanoparticles with PEG-2kDa vs. 4 kDa coatings, we demonstrated that PEG-4kDa AuNPs showed prolonged blood circulation and reduced liver accumulation in mice. These findings align with observations from chemically synthesized nanoparticles. The study [41] compared biodistribution of AuNPs coated with PEG-750Da and 10 kDa in healthy rats after intravenous administration. PEG-10kDa AuNPs showed significantly longer circulation, with 18% still detected in blood after 24 h compared to ~0.4% for PEG-750Da.

The study by Perrault et al. [42] systematically investigated the bloodstream half-life of AuNPs with core diameters ranging from 18 to 87 nm and PEG molecular weights of 2, 5, and 10 kDa. Following intravenous injection to mice, half-life was inversely proportional to core size, as expected. However, for particles of identical core size, those coated with higher molecular weight PEG exhibited longer circulation times. This is because longer PEG chains provide a more effective shield against opsonization and phagocytosis, thereby delaying elimination. Thus, a key conclusion of our work is the demonstration of fundamental analogy in behavior between chemically synthesized and laser-ablated AuNPs, confirming the universality of previously established principles.

Based on our data, we can formulate the following recommendations for the use of AuNPs as contrast agents in preclinical research. PEGylated AuNPs are advisable for imaging the heart and blood vessels, with an optimal time window of up to 24 h post-injection. They are also suitable for visualizing subcutaneous tumors. A limitation of this study is that tumor enhancement was investigated only for the PEG-4kDa coating, but the obtained pharmacokinetic data suppose that PEG-2kDa would also be sufficient for tumor accumulation. For imaging the abdominal organs (liver and spleen), BSA-AuNPs are more practical. They rapidly accumulate in these organs, providing intense contrast enhancement in the liver from 5 min and in the spleen from 1 h post-injection.

Another notable finding was the preferential accumulation of PEG-4kDa AuNPs in the stromal elements of the Ca755 tumor—specifically within the fibrous septa and pseudocapsule—compared to the parenchymal component. This observation aligns with the typical accumulation pattern of nanoparticles in solid tumor nodules reported in the literature [43]. This feature may offer utility in preclinical research. The contrast enhancement of the pseudocapsule can facilitate the visual delineation of the tumor margin from surrounding healthy tissue, and the enhancement of fibrous septa enables a detailed analysis of the internal tumor architecture. Maximum contrast enhancement throughout the tumor tissue was observed within a time window of 5 to 48 h. However, the retention of nanoparticles within the pseudocapsule allowed for visualization of the tumor boundaries for up to 6 days.

The functionalization strategy developed for bare laser-ablated gold nanoparticles can be extended to other laser-ablated nanostructures with an outer gold surface—such as Fe_3_O_4_@Au core–shell or core–satellite particles [44,45]. Such nanoparticles could serve as dual-mode contrast agents for both CT and MRI, significantly expanding imaging capabilities in preclinical research. The choice of coating would be determined by the required pharmacokinetic profile for a specific biomedical objective.

In this study, we focused exclusively on AuNPs as contrast agents for CT. However, the obtained pharmacokinetic and biodistribution data could also be valuable for other biomedical applications. Specifically, AuNPs of various structures are considered promising radiosensitizers [46,47,48], and laser-ablated nanoparticles may also serve this function [39]. Our data on the accumulation of AuNPs in tumor tissue could facilitate radiation therapy planning, as the optimal time interval between nanoparticle administration and irradiation onset depends on achieving peak gold concentrations within the tumor.

## 5. Conclusions

This study successfully fabricated and evaluated three distinct contrast agents from a single stock of laser-ablated AuNPs, functionalized with PEG-2kDa, PEG-4kDa, or lipoic acid with BSA coatings. Our findings demonstrate that the surface coating is the primary determinant of the agents’ pharmacokinetic behavior and thus their suitability for specific preclinical imaging applications. Particularly, PEGylated AuNPs are recommended for cardiovascular imaging, providing a practical time window of up to 24 h post-injection, and for subcutaneous tumor (Ca755), from 5 to 48 h post-injection. Conversely, BSA-coated AuNPs are optimal for imaging of abdominal organs, providing intense contrast enhancement in the liver from 5 min and in the spleen from 1 h post-injection.

A particularly significant result was the selective accumulation of PEGylated AuNPs in the stromal elements of Ca755 tumor, specifically within fibrous septa and the pseudocapsule, rather than the parenchyma. This feature enables two key applications: precise delineation of the tumor margin from healthy tissue and detailed analysis of the internal tumor architecture. The prolonged retention of contrast in the pseudocapsule, lasting up to 6 days, underscores the potential of these agents for longitudinal studies without repeated administration.

## Figures and Tables

**Table 1:** Physical and chemical characteristics of AuNPs.

Parameter	PEG-4kDa AuNPs	PEG-2kDa AuNPs	BSA-AuNPs
Core size (mean ± SD), nm	6.0 ± 1.4	5.8 ± 1.8	5.0 ± 1.3
Hydrodynamic size (mean ± FWHM), nm	41 ± 18	13 ± 14	9 ± 7
ζ-potential (mean ± FWHM), mV	−31 ± 17	−26 ± 11	−25 ± 16
Peak of plasmon resonance, nm	506	506	508
Concentration (mean ± SD), mg Au/mL	76 ± 3	83 ± 2	65 ± 5

## Data Availability

The data are included within the article and Supplementary Materials.

## Supplementary Materials

The following supporting information can be downloaded at: https://www.mdpi.com/article/10.3390/nano15241851/s1, Figure S1. CT radiodensity calibration: (a) CT image of calibration phantom, tubes contain water and colloid solutions of AuNPs with gold concentration from 1.3 to 25.5 mg Au/mL; (b) calibration plot. Figure S2. EDX spectra of AuNPs with different coatings: (a) PEG-2kDa; (b) PEG-4kDa; (c) BSA. Figure S3. FTIR spectra of AuNPs with different coatings: (a) PEG-2kDa attached to the gold surface via SH-group; (b) PEG-4kDa conjugated via lipoic acid; (c) BSA conjugated via lipoic acid. Figure S4. Hydrodynamic size distribution of AuNPs: uncoated; coated with only lipoic acid (AuNP-LA); coated with lipoic acid and stabilized with BSA (AuNPs BSA-LA). Table S1. Hydrodynamic size (mean ± FWHM) of AuNPs: uncoated; coated with only lipoic acid (AuNPs-LA); coated with lipoic acid and stabilized with BSA (AuNPs BSA-LA). Figure S5. SEM images: (a) PEG-2kDa AuNPs; (b) PEG-4kDa AuNPs; (c) BSA-AuNPs. Figure S6. Healthy BALB/c mouse (pre-injection) and mouse with skin discoloration after AuNP injection. Figure S7. Dynamics of gold concentration: (a) blood; (b) liver tissue; (c) spleen tissue. Concentrations were quantified based on CT radiodensity changes using a calibration curve derived from phantom studies. Figure S8. CT images of mouse kidneys and bladder acquired 1 h post injection of nanoparticles with three distinct coatings: (a) kidneys, coronal view, yellow arrows indicate the proximal ureter; (b) bladder, axial view, marked with yellow rectangle. Figure S9. Dynamic CT images of liver and spleen (coronal view) acquired up to 6 months post injection of PEG-2kDa AuNPs, PEG-4kDa AuNPs and BSA-AuNPs. No changes in contrast enhancement pattern were observed from 7 days to 6 months post injection.

## Abbreviations

The following abbreviations are used in this manuscript:

CT	Computed tomography
ML	Machine learning
AI	Artificial Intelligence
EPR	Enhanced permeability and retention
AuNPs	Gold nanoparticles
PEG	Polyethylene glycol
BSA	Bovine serum albumin
ICP-OES	Inductively coupled plasma optical emission spectrometry
DLS	Dynamic light scattering
SEM	Scanning electron microscopy
