# Supplementary material for: Laser-Ablated Gold Nanoparticles as Tunable Contrast Agents for Preclinical Imaging
Source: Nanomaterials (Basel). 2025 Dec 10;15(24):1851. doi: 10.3390/nano15241851 (PMC12736187; doi:10.3390/nano15241851)
Supplement: Supplementary file 1 [file nanomaterials-15-01851-s001.zip › nanomaterials-3985809-supplementary.pdf]

Laser-ablated gold nanoparticles as tunable contrast agents for preclinical imaging

Supplementary

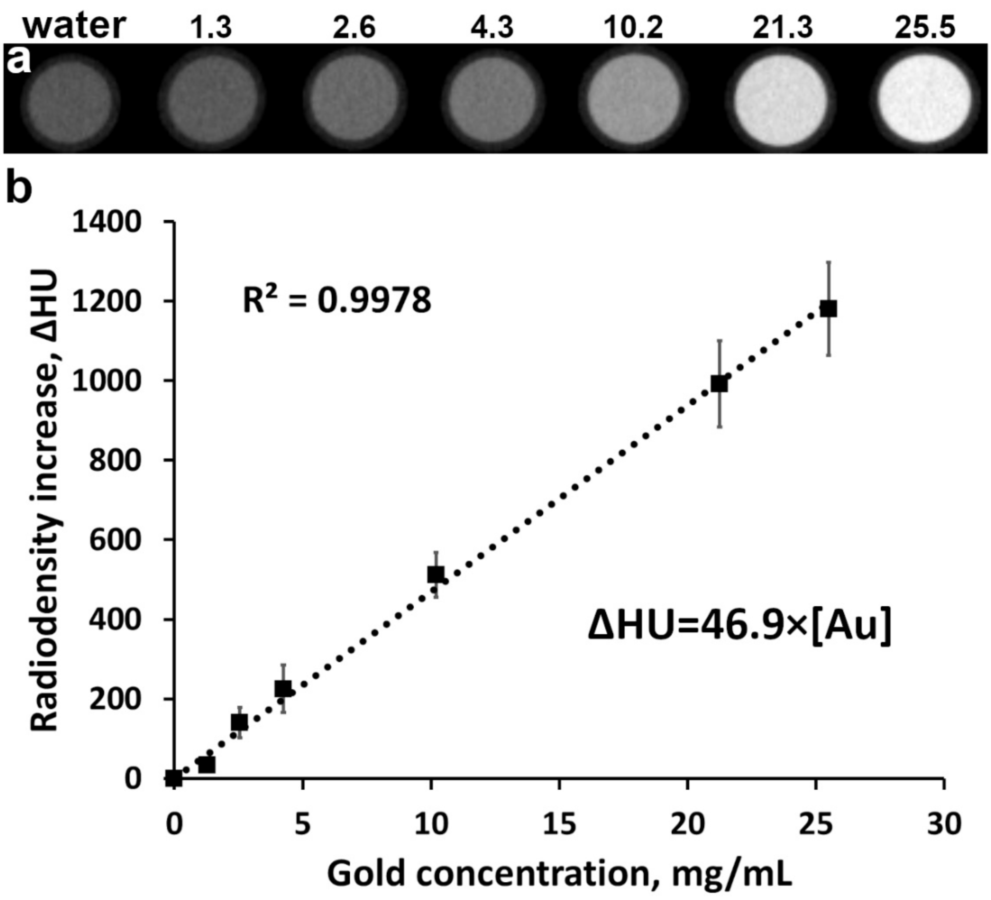

Figure S1. CT radiodensity calibration: (a) CT image of calibration phantom, tubes contain water and colloid solutions of AuNPs with gold concentration from 1.3 to 25.5 mg Au/mL; (b) calibration plot.

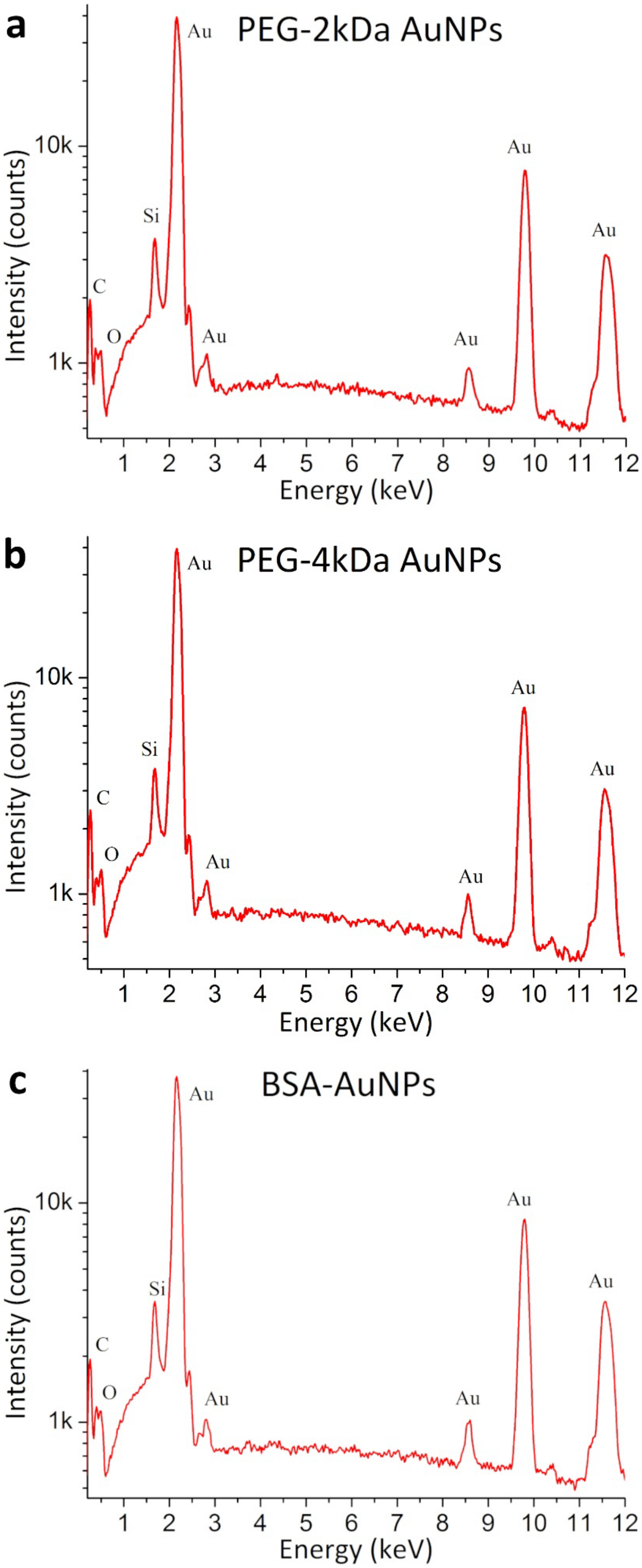

Figure S2. EDX spectra of AuNPs with different coatings: (a) PEG-2kDa; (b) PEG-4kDa; (c) BSA.

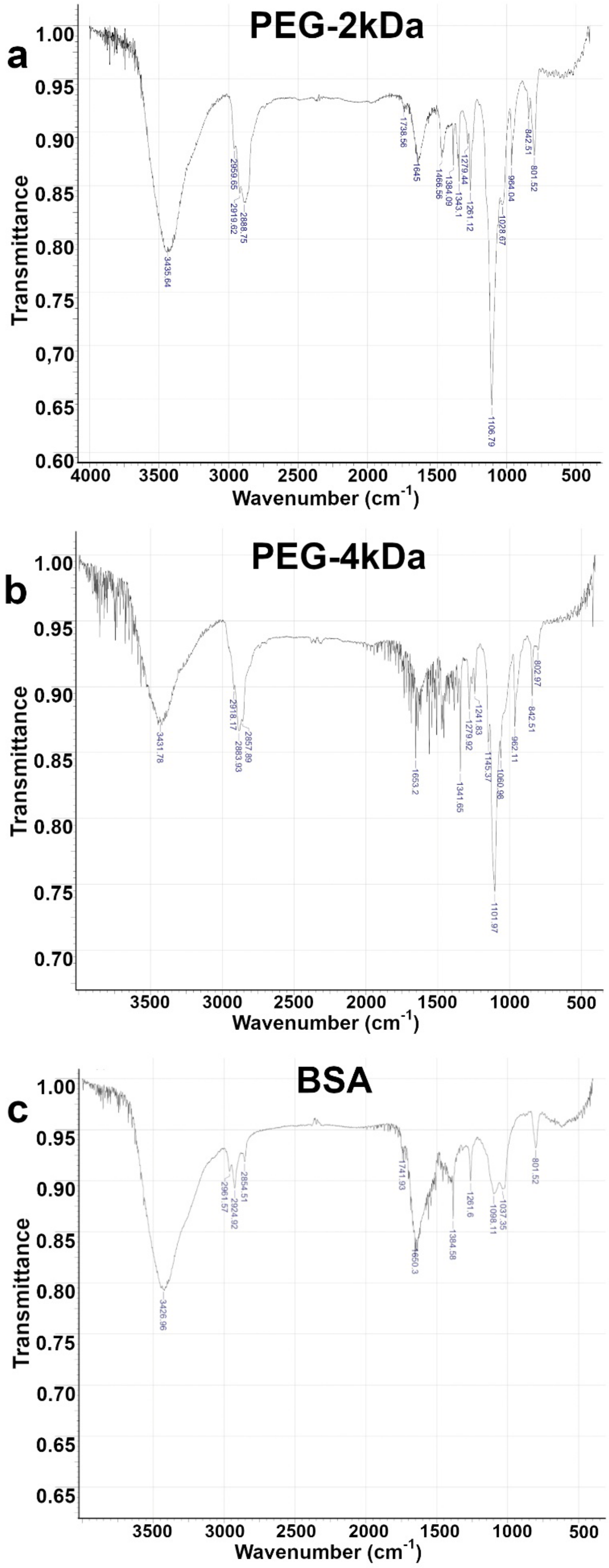

Figure S3. FTIR spectra of AuNPs with different coatings: (a) PEG-2kDa attached to the gold surface via SH-group; (b) PEG-4kDa conjugated via lipoic acid; (c) BSA conjugated via lipoic acid.

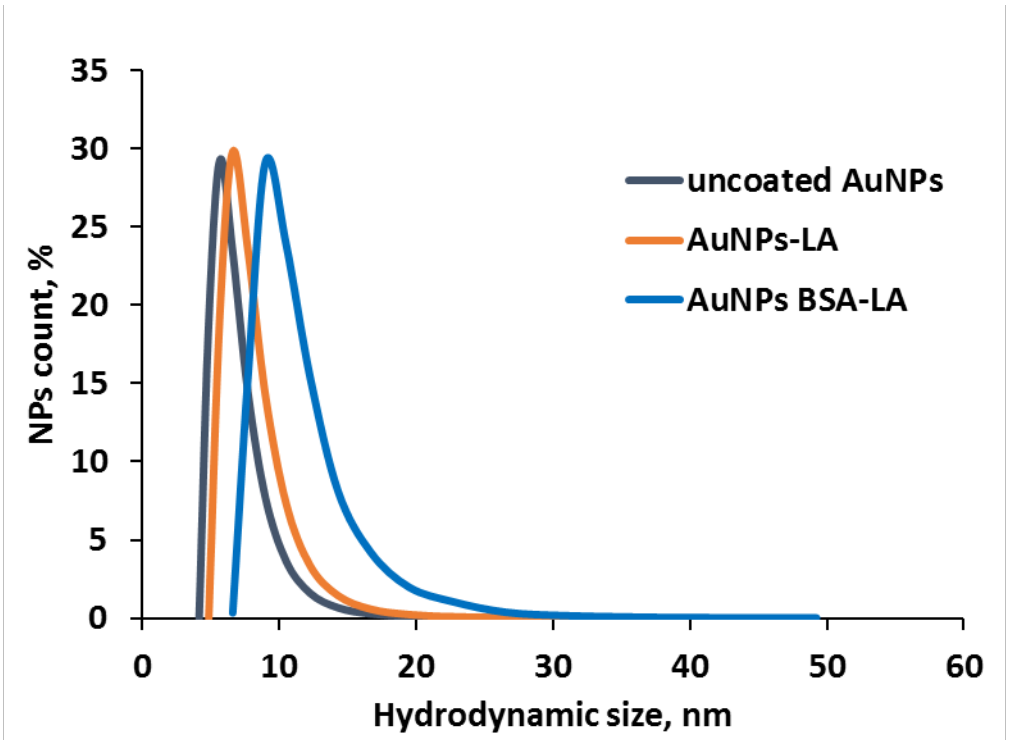

Figure S4. Hydrodynamic size distribution of AuNPs: uncoated; coated with only lipoic acid (AuNP-LA); coated with lipoic acid and stabilized with BSA (AuNPs BSA-LA).

Table S1. Hydrodynamic size (mean±FWHM) of AuNPs: uncoated; coated with only lipoic acid (AuNPs-LA); coated with lipoic acid and stabilized with BSA (AuNPs BSA-LA).

Parameter	uncoated AuNPs	LA-AuNPs	AuNPs BSA-LA
Hydrodynamic size (mean±FWHM), nm	6±4	7±5	9±7

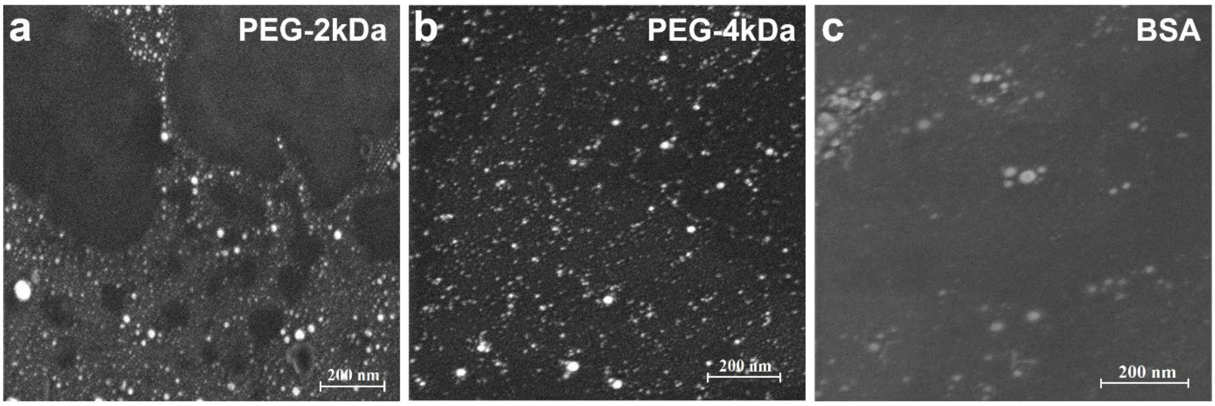

Figure S5. SEM images: (a) PEG-2kDa AuNPs; (b) PEG-4kDa AuNPs; (c) BSA-AuNPs.

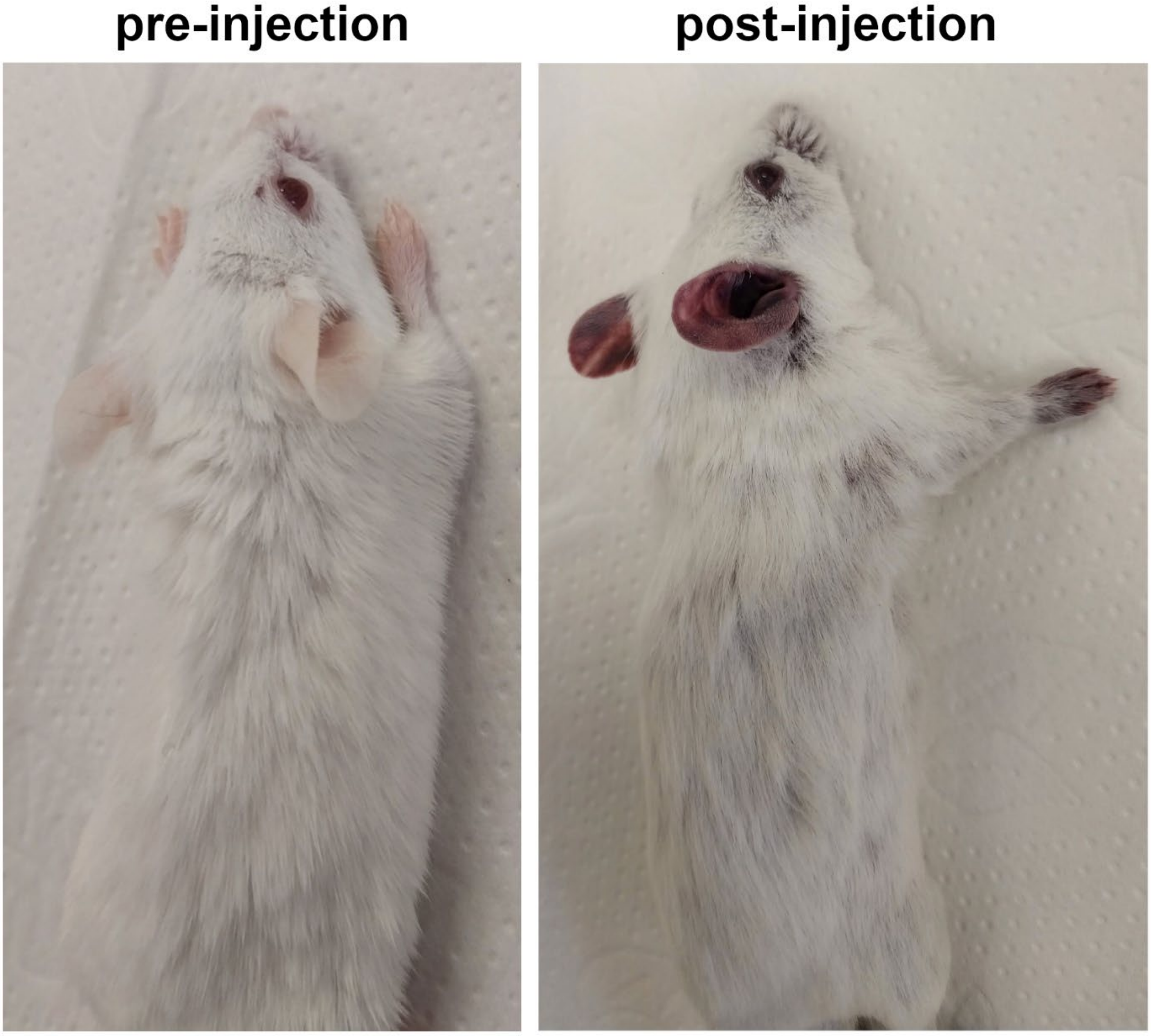

Figure S6. Healthy BALB/c mouse (pre-injection) and mouse with skin discoloration after AuNP injection.

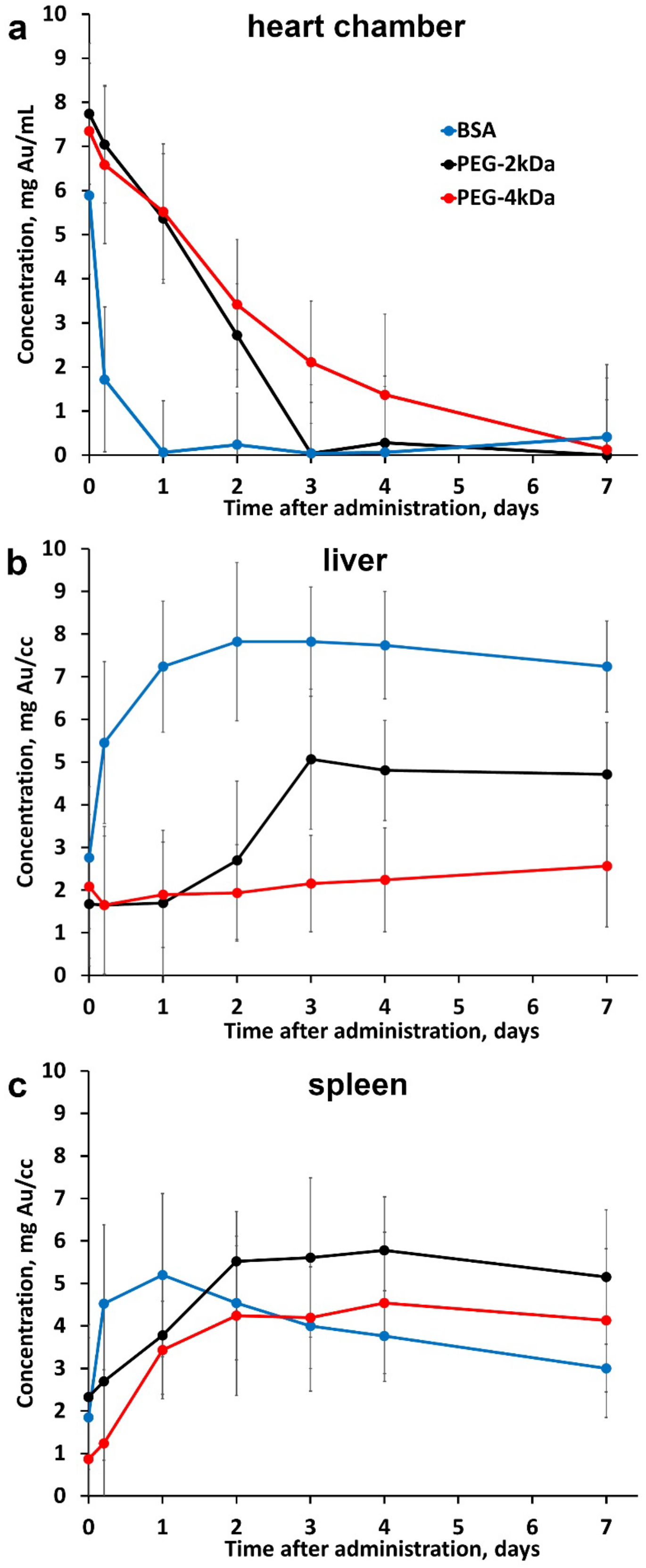

Figure S7. Dynamics of gold concentration: (a) blood; (b) liver tissue; (c) spleen tissue. Concentrations were quantified based on CT radiodensity changes using a calibration curve derived from phantom studies.

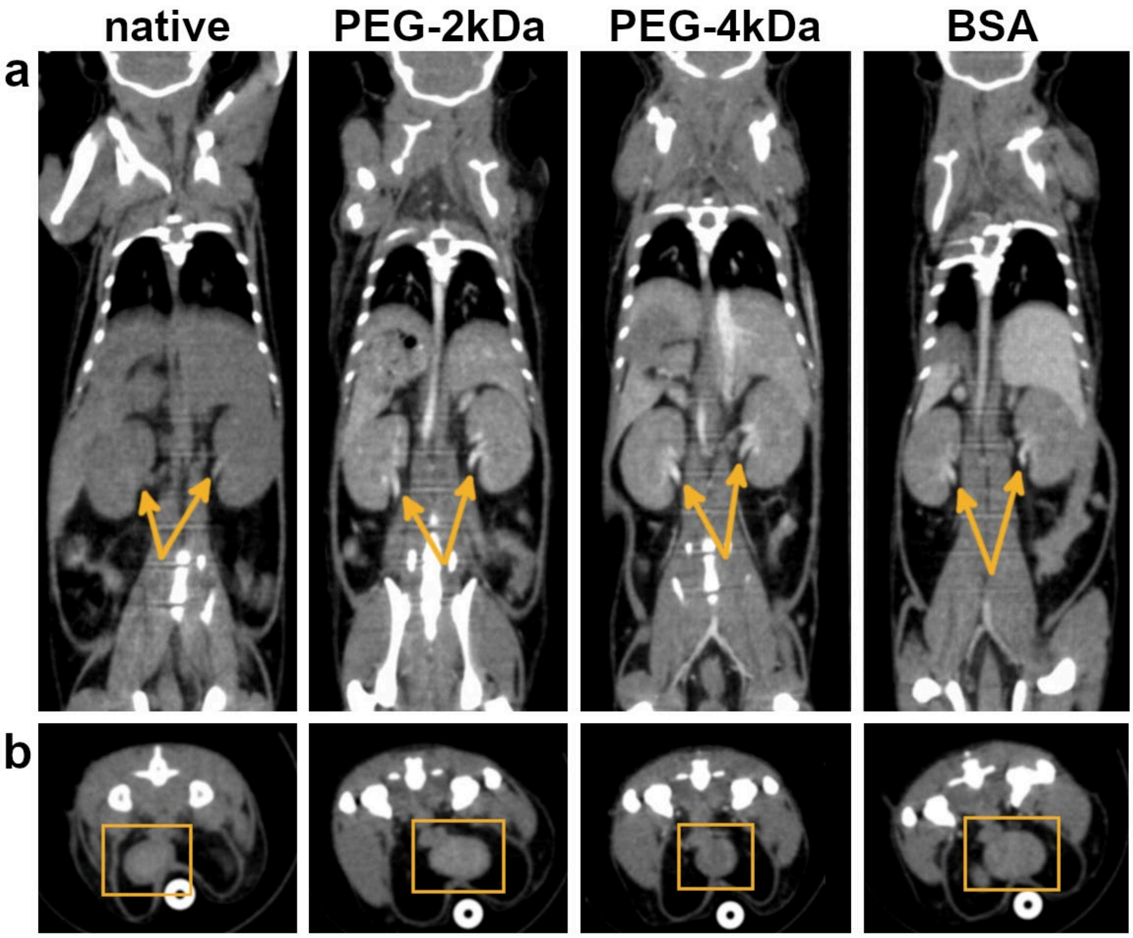

Figure S8. CT images of mouse kidneys and bladder acquired 1 h post injection of nanoparticles with three distinct coatings: (a) kidneys, coronal view, yellow arrows indicate the proximal ureter; (b) bladder, axial view, marked with yellow rectangle.

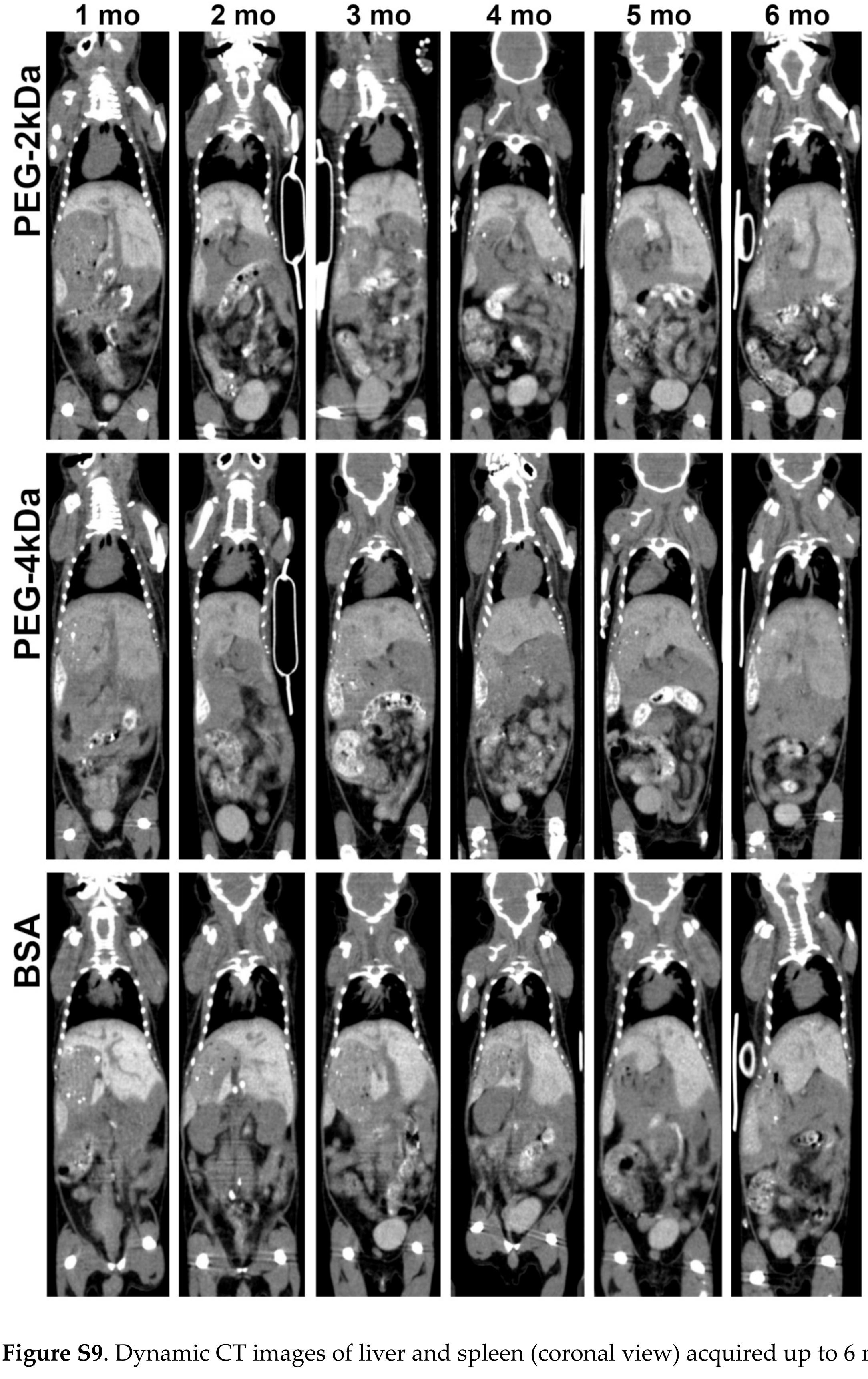

Figure S9. Dynamic CT images of liver and spleen (coronal view) acquired up to 6 months post injection of PEG-2kDa AuNPs, PEG-4kDa AuNPs and BSA-AuNPs. No changes in contrast enhancement pattern were observed from 7 days to 6 months post injection
